# Positive plasma cotinine during platinum-based chemotherapy is associated with poor response rate in advanced non-small cell lung cancer patients

**DOI:** 10.1371/journal.pone.0219080

**Published:** 2019-07-01

**Authors:** Philippine Dacosta-Noble, Adrien Costantini, Coraline Dumenil, Jennifer Dumoulin, Pierre Helly de Tauriers, Violaine Giraud, Sylvie Labrune, Jean-François Emile, Jean-Claude Alvarez, Thierry Chinet, Etienne Giroux Leprieur

**Affiliations:** 1 Department of Respiratory Diseases and Thoracic Oncology, APHP-Hopital Ambroise Paré, Boulogne-Billancourt, France; 2 EA 4340, UVSQ, Université Paris-Saclay, Boulogne-Billancourt, France; 3 Centre de Ressources Biologiques, APHP-Hopital Ambroise Paré, Boulogne-Billancourt, France; 4 AP-HP, Hôpital Raymond Poincaré, Service de Pharmacologie Toxicologie, INSERM U-1173, UVSQ, Université Paris-Saclay, Garches, France; University of South Alabama Mitchell Cancer Institute, UNITED STATES

## Abstract

**Introduction:**

Patients with advanced non-small cell lung cancer (NSCLC) are most of the time treated with a first-line cytotoxic chemotherapy. Tobacco use is responsible for 90% of lung cancer. The aim of this study was to evaluate the impact of smoking continuation during first-line chemotherapy on tumor response in advanced-stage NSCLC.

**Materials and methods:**

All patients with an advanced-stage NSCLC (IIIb or IV), treated with first-line platinum-based chemotherapy in our Department between June 2013 and July 2017 were included. Smoking status was assessed at inclusion by self-report, then at the tumor assessment consultation after 2 months of treatment, by both self-report and plasmatic cotinine measurement. Chemotherapy response, progression-free survival (PFS), overall survival (OS) and stage 3–4 toxicity were registered.

**Results:**

Ninety-seven patients were included: 8 (8%) declared to be non-smokers, 56 (58%) current smokers and 33 (34%) former smokers at diagnosis. At the first tumor evaluation, 24 (25%) self-reported as active smokers and 73 (75%) as non-smokers; overall response rate (ORR) was respectively 38% and 48% (p = 0.373). Fifty-four patients had a plasmatic cotinine evaluation at the first tumor evaluation. Seventeen patients (32%) had a positive cotinine rate (median 108ng/mL, IQR 31–236). Six patients (35%) had positive cotinine rate whereas declaring to be non-smokers at the first tumor evaluation. ORR was 18% in case of positive cotinine rate, and 57% when negative (p = 0.007). Regardless of the method for smoking status evaluation, PFS, OS and grade 3–4 toxicities were similar between smoker and non-smoker patients at the first tumor evaluation.

**Conclusion:**

Smoking continuation during platinum-based chemotherapy, reflected by positive plasma cotinine rate, was associated with a poor ORR.

## Introduction

Lung cancer is the leading cause of cancer-related death worldwide [1]. Despite many progress regarding treatments, the overall five-year survival rate remains low at around 15% [1]. The majority of these tumors are non-small cell lung cancer (NSCLC), often diagnosed at an advanced or metastatic stage with a five-year survival rate of 5% [1]. When patients do not present with an oncogenic addiction or with a high level of Programmed Death Ligand 1 (PD-L1) expression, treatment strategy of metastatic NSCLC relies on platinum-doublet cytotoxic chemotherapy (CT), representing around 2/3 of the cases [2]. Smoking is responsible for 90% of lung cancers. There is a close relation between the level of smoking and the risk of developing lung cancer [1]. Most patients with lung cancer have a history of cigarette consumption and 20% to 40% of them are active smokers at the diagnosis of lung cancer [3–5]. Despite help and encouragements to quit smoking, the pursuit of active cigarette consumption after the diagnosis of lung cancer remains a major issue. Between 30% and 80% of smokers continue to smoke after the diagnosis of lung cancer, all stages of disease considered [4,6,7]. Studies have analysed the impact of continued smoking in lung cancer, especially in localised operable disease. In case of thoracic surgery, continued tobacco intoxication leads to an increase in the risk of general complications, in particular infectious, coronary and respiratory complications such as broncho-pulmonary fistula, and a higher rate of hospital and ICU admissions [8,9]. Furthermore, in advanced NSCLC harbouring an *Epidermal Growth Factor Receptor* (EGFR) mutation, it has been shown that smoking causes a decreased efficacy of EGFR tyrosine kinase inhibitors (TKIs) [10,11]. However, few studies have analysed the impact of smoking on tumor response, CT toxicity and survival in advanced NSCLC treated with first-line CT.

The aim of this study was to evaluate the impact of continued smoking during platinum-based doublet cytotoxic CT in advanced NSCLC on tumor response, grade 3–4 toxicities, progression free survival (PFS) and overall survival (OS). Evaluation of smoking cessation was based on patients’ oral declaration and on systematic sampling of plasmatic cotinine at first tumor evaluation at two months.

## Materials and methods

### Endpoints

The primary endpoint was to evaluate in an exploratory manner tumor response depending on the pursuit of smoking (evaluated by patients’ oral declaration and measure of plasmatic cotinine) in patients with advanced or metastatic NSCLC treated with platinum-doublet cytotoxic CT. The secondary endpoints were to evaluate PFS, OS and grade 3–4 toxicities.

### Patients

We included consecutive patients with advanced or metastatic NSCLC treated with first line platinum-based doublet cytotoxic CT in the Respiratory and Thoracic Oncology Department of an Academic Hospital (APHP-Hopital Ambroise Paré, Boulogne-Billancourt, France) between June 2013 and July 2017. The study was both retrospective and prospective, with a retrospective inclusion period between June 2013 and March 2016 and a prospective inclusion period from March 2016 to July 2017. The inclusion criteria were the following: patients with histologically proven stage IIIb (not amenable to radiotherapy) or IV NSCLC who had received first line treatment with platinum-based doublet cytotoxic CT. Exclusion criteria were the following: first-line targeted therapy (EGFR or Anaplastic Lymphoma Kinase (ALK) TKIs) or curative thoracic radiotherapy (66 Grays or more). Clinical and pathological features were extracted from patients’ charts: demographic features, smoking status before and during treatment, disease stage (according to the 2009 TNM classification), histological subtype, date of diagnosis, medical history, Performance Status (PS) according to the Eastern Cooperative Oncology Group (ECOG), treatments received, response to treatment according to RECIST criteria v1.1 [12], treatment toxicity (according to CTCAE 4.0 criteria), PFS and OS. Progression date, date of death and date of last news (last hospital visit) were collected. Cut-off date was the 4^th^ of June 2018. Tumor response, according to RECIST v1.1 criteria, was evaluated every two months by clinical examination and brain-thoracic-abdominal and pelvic computed tomography scanning. CT response was evaluated by a radiologist specialised in thoracic imaging and each case was discussed and validated during a multi-disciplinary meeting. Overall response rate was defined as the proportion of patients with complete or partial response.

### Smoking status

Smoking status of each patient was evaluated at diagnosis and at first tumor evaluation after two cycles of CT. Smoking status was registered in the patient’s file based on the oral declarations of each patient: smoker or non-smoker, date of smoking cessation, amount smoked in pack-years (PY). At diagnosis, non-smokers were defined as having smoked less than 100 cigarettes in their lifetime. Former smokers were defined as having stopped smoking at least one year before the diagnosis of NSCLC. Smokers were defined as actively smoking in the year before the diagnosis of NSCLC. At first tumor evaluation, we re-evaluated each patient’s smoking status: still non-smoker, still smoker or smoking cessation. Patients were then grouped in two groups: still smoker or non-smoker (still non-smoker + smoking cessation). The use of nicotine substitutes was also noted.

### Plasmatic cotinine

We performed plasmatic cotinine measurements at first tumor evaluation after two months of treatment. Plasma was collected prospectively after signature of a consent form. Cotinine was measured using a liquid chromatographic technique coupled with mass spectrometry (Toxicology department, APHP-Hôpital Raymond Poincaré). The limit of quantification of the LC/MS/MS method was 2 ng/mL.

### Statistical analysis

The current study was an exploratory study, thus, there was no statistical hypothesis or a minimum number of patients to include. Comparison of clinical characteristics between the patient groups was performed using the Mann-Whitney test or Fisher’s exact test depending on the distribution of the variables. Categorical comparisons were performed using the Chi2 test. Evaluation of PFS and of OS was performed using the log-rank test (Kaplan-Meier method). Statistical analysis was performed using XL STAT 2018 (Addinsoft). A p-value inferior to 0.05 was considered as significant. Anonymized study database can be found as S1 File.

### Ethical considerations

This study was approved by the Institutional Review Board (CEPRO) of the Société de Pneumologie de Langue Française (SPLF) on the 16^th^ of April 2016 (number 2016–011). Patients who underwent cotinine measurement had signed a consent form beforehand (approval by the Comité de Protection des Personnes (CPP) Ile-de-France n°VIII). Data were fully anonymized before the authors accessed them.

## Results

### Patients’ characteristics

Between June 2013 and July 2017, 160 patients were treated in the Department for stage IIIb (not amenable to radiotherapy) or IV NSCLC. Out of these 160 patients, 16 had EGFR mutation, 3 had ALK rearrangement and 44 did not receive systemic treatment. Ninety-seven patients were treated with platinum-based CT and were included in the study. Among them, seventy-two patients (74%) were retrospectively included between June 2013 and March 2016 and 25 patients (26%) were prospectively included between March 2016 and July 2017. Patients’ characteristics are shown in Table 1. The study population included 66% of men with a median age of 68 years (range 63–73). The most frequent histological subtype was adenocarcinoma (65%, n = 53). Twenty-seven patients (28%) were treated with cisplatin-based doublet CT and 70 (72%) with carboplatin-based doublet CT. The drug most frequently associated with platinum CT was pemetrexed (66%, n = 64) and paclitaxel (20%, n = 19). At cancer diagnosis, eight patients (8%) declared themselves as non-smokers, 56 patients (58%) as current smokers and 33 (34%) as former smokers.

**Table 1:** Characteristics of the overall population at the first tumor evaluation, with smoking status evaluated by patient’s declaration.

	Total (N = 97)	Still smoker (N = 24)	Non-smoker (N = 73)	p-value*
Age (median (range))	68.1 (43.0–86.9)	66.7 (49.6–78.3)	68.4 (43.0–86.9)	0.396
Male	64 (66.0)	10 (41.7)	54 (78.1)	0.004
Female	33 (34)	14 (58.3)	19 (21.9)	
Never smoker	8 (8.2)	0 (0.0)	8 (11.0)	
Pack-year (median (range))	40 (0–110)	35 (10–110)	40 (0–90)	0.837
PS				0.471
0–1	81 (83.5)	21 (87.5)	60 (82.2)	
2	16 (16.5)	3 (12.5)	13 (17.8)	
Histology				0.980
Adenocarcinoma	63 (64.9)	16 (66.6)	47 (64.4)	
Squamous cell carcinoma	17 (17.5)	4 (16.7)	13 (17.8)	
Other	17 (17.5)	4 (16.7)	13 (17.8)	
Stage 4	92 (94.8)	21 (87.5)	71 (97.3)	0.061
Kras mutation	22 (22.7)	5 (20.8)	17 (23.3)	0.803
Platinum drug				0.721
Cisplatin	27 (27.8)	6 (25.0)	21 (28.8)	
Carboplatin	70 (72.2)	18 (75.0)	52 (71.2)	
Associated drug				0.635
Pemetrexed	64 (66.0)	18 (75.0)	46 (63.0)	
Vinorelbine	4 (4.1)	1 (4.2)	3 (4.1)	
Gemcitabine	6 (6.2)	0 (0.0)	6 (8.2)	
Paclitaxel	19 (19.6)	4 (16.7)	15 (20.5)	
Etoposide	2 (3.1)	1 (4.2)	1 (1.4)	
bevacizumab	12 (12.4)	4 (16.7)	8 (11.0)	
Grade 3–4 toxicity	45 (46.4)	11 (45.8)	34 (46.6)	0.950
Dose reduction	14 (14.4)	3 (12.5)	11 (15.1)	0.553

*p-value calculated by Chi^2^ test (except for age: Mann-Whitney-test).

Values are expressed (if no otherwise specified) as n (%).

Amongst the 56 current smokers, 24 declared to continue smoking at first tumor evaluation (43%) and 32 declared having stopped smoking (57%). Patients who declared keeping on smoking were mostly women (58%) with a median age of 68 years (range 61–72) and a median of 40 PY (range 30–53). Patients who had declared smoking cessation used nicotine substitutes in seven cases (27%).

There was no difference in term of CT dose reduction (due to toxicities) between patients who declared continued smoking and the other patients (13% in the group with continued smoking vs 15% in the non-smoker group, p = 0.553).

### Characteristics of the population having undergone cotinine measurement

Forty-three patients refused to sign the consent form and the plasma collection, and 54 underwent plasmatic cotinine measurement at first tumor evaluation after two cycles of CT. There was no statistical difference concerning the patients’ characteristics when comparing this group of patients and the group of patients who declined the plasma collection.

Cotinine measurement was negative in 37 patients (69%) and positive in 17 patients (31%). Median cotinine level in case of positive measurement was 108ng/mL (IQR 31–236). The characteristics of these patients are presented in Table 2. The group of patients with positive plasmatic cotinine was made up of 12 women (71%) with a median age of 69 (range 61–74). Amongst these 17 patients, only 11 declared to be smokers, 5 reported having quit smoking since the diagnosis, one since a year, resulting in a difference between the patients’ declaration concerning smoking status and plasmatic cotinine levels in 6 cases (35%). Amongst the conflicting cases, four (67%) were women with a median age of 69 years (range 65–74) and a median tobacco consumption of 40 PY (range 30–51). Two patients with positive plasmatic cotinine declared using nicotine substitutes (gum and patch for one patient, patch alone for one patient, with respective cotinine levels of 300ng/mL and 241ng/mL). Four patients who declared using nicotine substitutes had negative plasmatic cotinine levels. There was no difference in term of CT dose reduction (due to toxicities) between patients with positive and negative plasmatic cotinine levels (6% in the positive cotinine group vs 16% in the negative cotinine group, p = 0.294).

**Table 2:** Characteristics of the population who underwent plasma tests at the first tumor evaluation, with smoking status evaluated by plasma cotinine.

	Total (N = 54)	Positive cotinine rate (N = 17)	Negative cotinine rate (N = 37)	p-value*
Age (median, range)	67.7 (43.0–86.9)	69.1 (49.6–78.3)	67.5 (43.0–86.9)	0.794
Male	32 (59.3)	5 (29.4)	27 (73.0)	0.002
Female	22 (40.7)	12 (70.6)	10 (27)	
Never smoker	2 (3.7)	0 (0.0)	2 (5.4)	
Pack-year (median (range))	40 (0–100)	30 (15–100)	40 (0–80)	0.325
PS				
0–1	47 (87.0)	14 (82.4)	33 (89.2)	0.487
2	7 (13.0)	3 (17.6)	4 (10.8)	
Histology				
Adenocarcinoma	38 (70.4)	13 (76.5)	25 (67.6)	0.445
Squamous cell carcinoma	8 (14.8)	3 (17.6)	5 (13.5)	
Other	8 (14.8)	1 (5.9)	7 (18.9)	
Stage 4	51 (94.4)	17 (100)	34 (91.9)	0.227
Kras mutation	14 (25.9)	4 (23.5)	10 (27.0)	0.785
Platinum drug				
Cisplatin	16 (29.6)	3 (17.6)	13 (35.1)	0.191
Carboplatin	38 (70.4)	14 (82.4)	24 (64.9)	
Associated drug				0.621
Pemetrexed	37 (68.5)	12 (70.6)	25 (37.6)	
Vinorelbine	4 (7.4)	1 (5.9)	3 (8.1)	
Gemcitabine	3 (5.6)	1 (5.9)	2 (5.4)	
Paclitaxel	9 (16.7)	2 (11.8)	7 (18.9)	
Etoposide	1 (1.9)	1 (5.9)	0 (0.0)	
Grade 3–4 toxicity	27 (50.0)	7 (41.2)	20 (54.1)	0.379
Dose reduction	7 (13.0)	1 (5.9)	6 (16.2)	0.294

*p-value calculated by Chi^2^ test (except for age: Mann-Whitney-test).

Values are expressed (if no otherwise specified) as n (%).

### Response to CT

**Fig 1:**
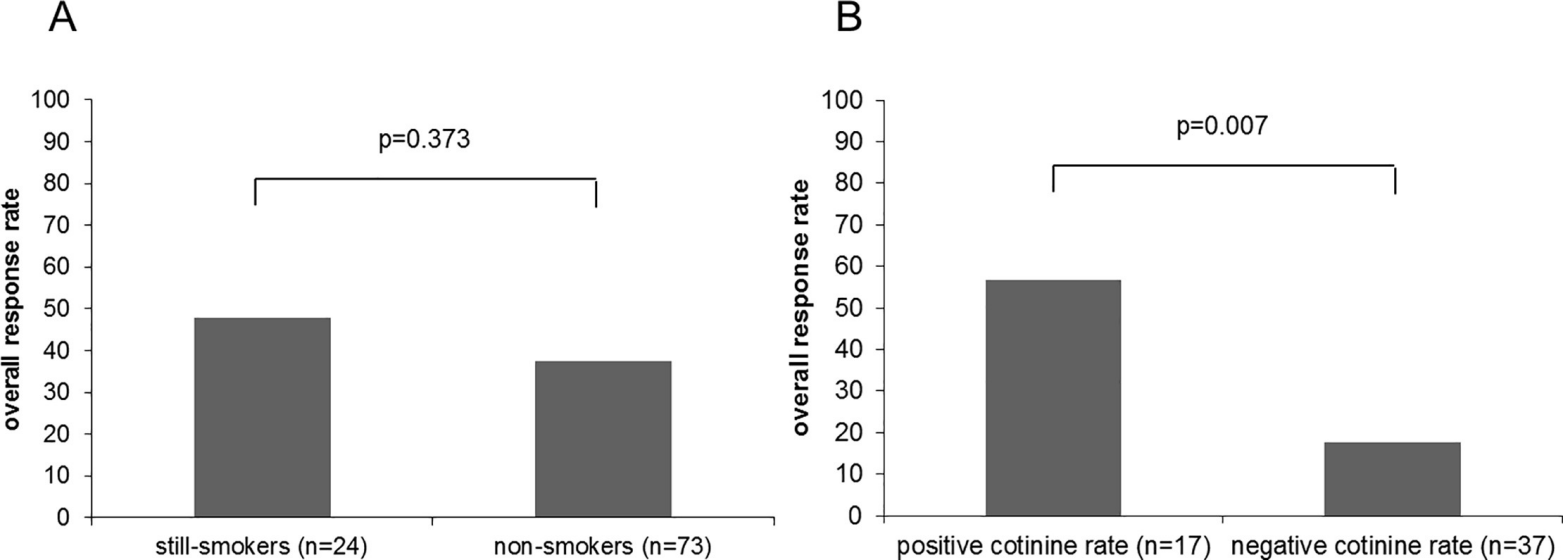
**Overall response rate according to smoking continuation evaluated at first tumor evaluation on patient’s declaration (A) or cotinine rate (B)**. p-value calculated by Chi^2^ test.

In the general population, the objective response rate (ORR) was 45%. In patients who declared themselves as smokers at 2 months (still smokers), the ORR was 38% and it was 48% in patients who declared themselves as non-smokers at the time of first tumor evaluation (p = 0.373) (Fig 1A).

In the population of patients who underwent plasmatic cotinine measurement, ORR was 44%. In patients with positive plasmatic cotinine levels at 2 months, ORR was 18% compared to 57% in patients with negative plasmatic cotinine levels at first tumor evaluation (p = 0.007) (Fig 1B).

### PFS and OS

**Fig 2:**
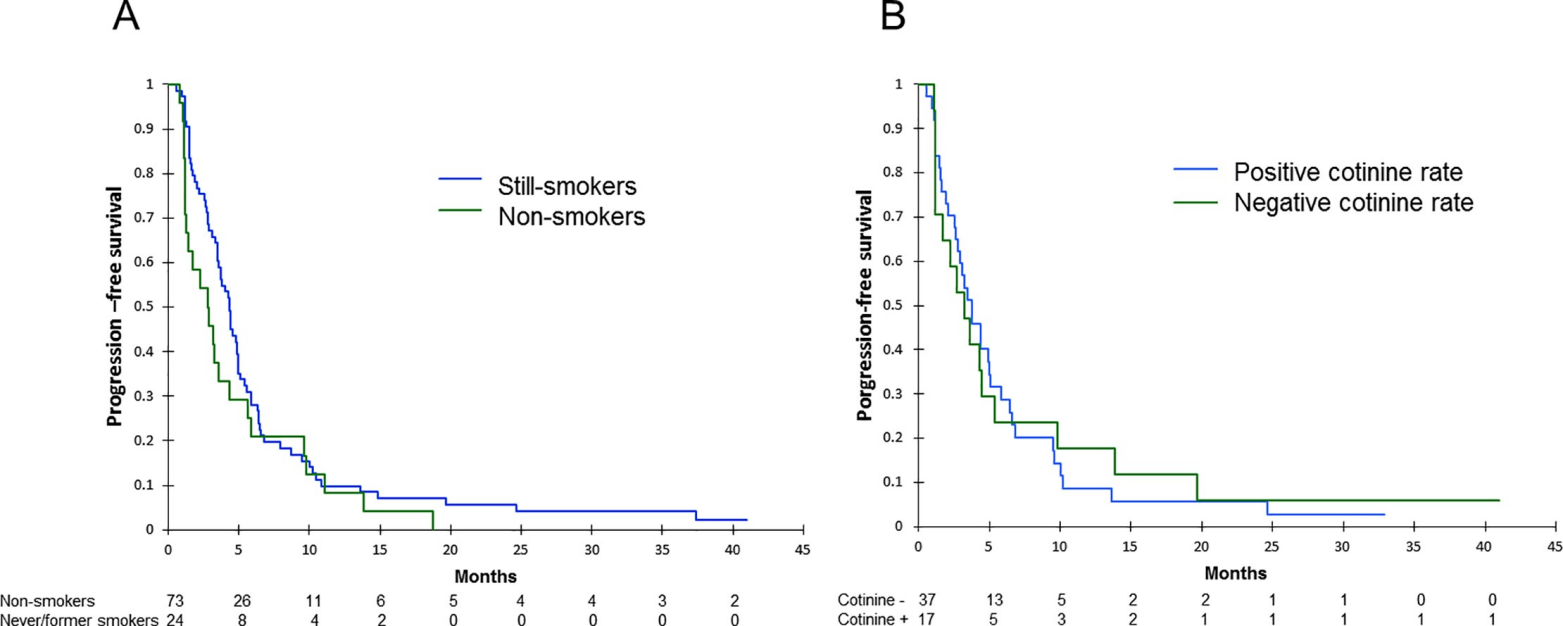
**Progression-free survival according to smoking continuation evaluated at first tumor evaluation on patient’s declaration (A) or cotinine rate (B).** p-value calculated by log-rank test.

There was no statistically significant difference in PFS between patients who were smokers (median of 2.2 months) and the others (median of 4.4 months) (p = 0.172) (Fig 2A). In the population who underwent plasmatic cotinine measurement, medians for PFS were 3.2 months and 3.7 months for patients with positive and negative plasmatic cotinine levels respectively (p = 0.919) (Fig 2B).

**Fig 3:**
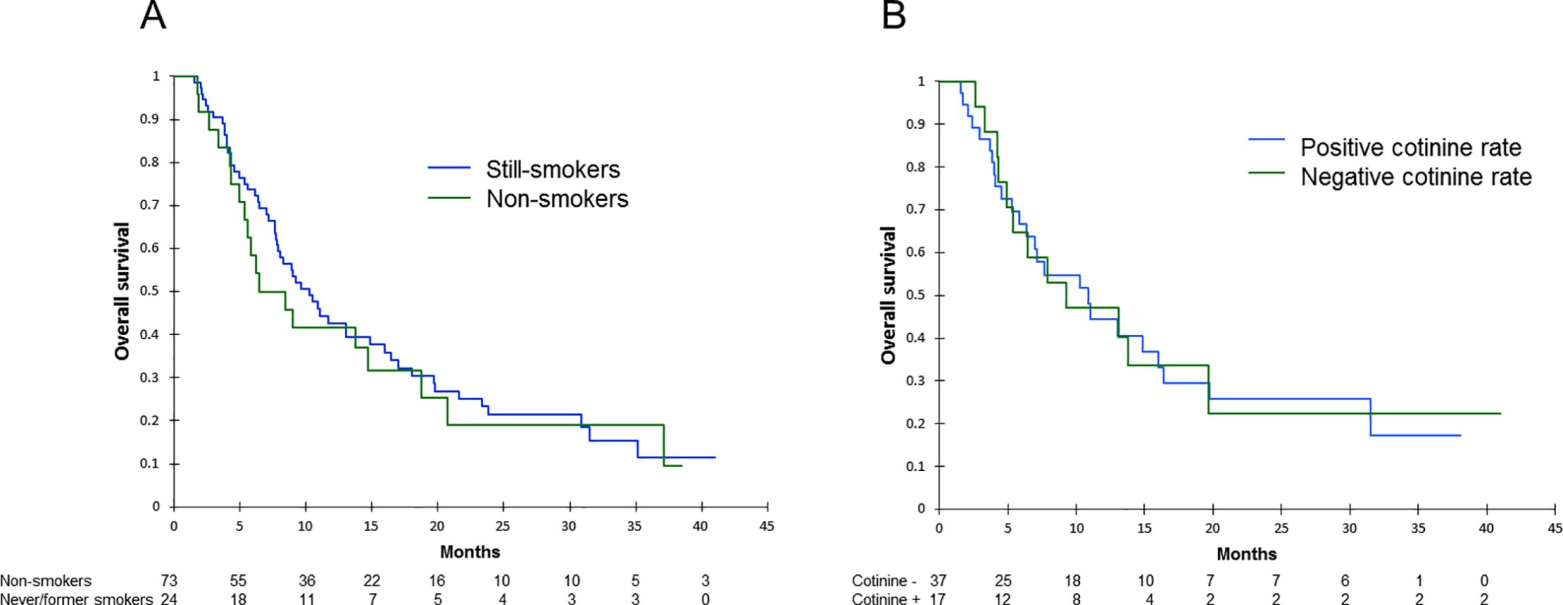
**Overall survival according to smoking continuation evaluated at first tumor evaluation on patient’s declaration (A) or cotinine rate (B).** p-value calculated by log-rank test.

In the overall population, median OS for those declaring themselves as smokers was 7.5 months and 10.3 months for those declaring themselves as non-smokers (p = 0.643) (Fig 3A). In the population who underwent plasmatic cotinine measurement, medians for OS were 9.2 months and 10.9 months for patients with positive and negative plasmatic cotinine levels respectively (p = 0.951) (Fig 3B).

### Grade 3–4 toxicity

In the overall population, there was no significant difference regarding the onset of grade 3–4 toxicity according to declared smoking status. Smokers had grade 3–4 toxicity in 46% and non-smokers in 47% of cases (p = 0.95). In the same way, the population who underwent plasmatic cotinine measurement, patients with positive plasmatic cotinine levels had grade 3–4 toxicity in 41% of cases compared to 54% in patients with negative plasmatic cotinine levels (p = 0.379) (Tables 3 and 4).

**Table 3:** Grade 3–4 toxicity profile according to smoking continuation, evaluated at first tumor evaluation on patient’s declaration.

	Total (N = 97)	Still smoker (N = 24)	Non- smoker (N = 73)
Fatigue	6 (6.2)	1 (4.2)	5 (6.8)
Nausea/Vomiting	6 (6.2)	1 (4.2)	5 (6.8)
Constipation	1 (1.0)	0 (0.0)	1 (1.4)
Anemia	20 (20.6)	4 (16.7)	16 (21.9)
Neutropenia	16 (16.5)	3 (12.5)	13 (17.8)
Thrombocytopenia	13 (13.4)	4 (16.7)	9 (12.3)
ALAT/ASAT increase	3 (3.1)	1 (4.2)	2 (2.7)
Cholestasis	4 (4.1)	1 (4.2)	3 (4.1)
Infection	9 (9.3)	1 (4.2)	8 (10.9)
Mucositis	4 (4.1)	3 (12.5)	1 (1.4)

Values are expressed as n (%).

**Table 4:** Grade 3–4 toxicity profile according to smoking continuation, evaluated at first tumor evaluation on cotinine rate.

	Total (N = 54)	Positive cotinine rate (N = 17)	Negative cotinine rate (N = 37)
Fatigue	0 (0.0)	0 (0.0)	0 (0.0)
Nausea/Vomiting	5 (9.3)	1 (5.9)	4 (10.8)
Constipation	0 (0)	0 (0)	0 (0)
Anemia	12 (22.2)	3 (17.6)	9 (24.3)
Neutropenia	10 (18.5)	3 (17.6)	7 (18.9)
Thrombocytopenia	9 (16.7)	3 (17.6)	6 (16.2)
ALAT/ASAT increase	2 (3.7)	1 (5.9)	1 (2.7)
Cholestasis	1 (1.8)	0 (0.0)	1 (2.7)
Infection	3 (5.5)	1 (5.9)	2 (5.4)
Mucositis	3 (5.5)	3 (17.6)	0 (0.0)

Values are expressed as n (%).

## Discussion

Our study has shown that continued smoking, reflected by positive plasmatic cotinine levels, during first-line CT was associated with decreased ORR (18% in case of positive plasmatic cotinine levels vs. 57% in case of negative plasmatic cotinine levels [p = 0.007]). In the overall population, 38% of patients who declared to continue smoking at 2 months had tumor response compared to 48% who declared themselves as non-smokers (p = 0.373). This inconsistency could be due to confusion bias when patients were classified as smoker and non-smokers according to their oral declarations: some patients would declare themselves as non-smokers when in fact they would continue smoking. Plasmatic cotinine measurement corrects this bias and gives us a correct patient classification.

No difference in PFS and OS was shown, be it using oral declarations or cotinine measurement. However, tumor response rate has been shown to be tightly associated with PFS and OS during CT [13,14]. The small size of our study with a lack of power for survival analyses might explain why we did not find differences concerning OS and PFS. Furthermore, some patients might have quit smoking later-on during treatment which could also have modified PFS and OS analyses in our study. At last, patients could have received different further treatment lines that could also influence OS. Using response as the primary endpoint frees us from this bias whilst remaining clinically relevant: reduction of tumor burden is not only associated with survival [13,14] but also with the improvement of symptoms due to cancer [15] as well of quality of life [16,17].

The results of our study regarding response to CT is consistent with data published by Tsao *et al* who compared the outcomes of smokers and non-smokers at cancer diagnosis and showing that smoking caused worse response to CT and shorter survival [18]. ORR was better in non-smokers (19%) than in former smokers (8%) or in current smokers (12%) (p = 0.004). Progression rates were lower in non-smokers (49%) than in former smokers (65%) and smokers (66%) (p = 0.002). One-year survival was higher in non-smokers (63%) than in former smokers (42%) or smokers (43%). However, the detail of smoking cessation after cancer diagnosis was not available in this study. Incriminating pursued smoking on decreased CT response rates after the initial diagnosis was thus not possible. Our study is innovating as it evaluated the impact of smoking continuation during CT on tumor response rates. Furthermore, in Tsao’s study, only 78% of patients received platinum-based CT which is now the recommended first-line therapy. Finally, the drug associated with platinum was not detailed, with association modalities that have evolved since 2006 with frequent use of pemetrexed in adenocarcinoma histological type. Duarte et al conducted a retrospective study in Brazil between 2000 and 2005 evaluating the impact of smoking on response to platinum-based CT [19]. Amongst the 285 patients, 63% were smokers, 27% were former smokers and 11% non-smokers. There was no significant different on tumor response between smokers and non-smokers. This study suggests that smoking more than 40 PY was the main predictor of negative response to CT (adjusted OR 10.42, CI 95% 5.13; 21.28). However, smoking habits were solely base on patients’ oral declarations. When evaluating response based on patients’ declarations we did not find a difference between the groups. Furthermore, the details of smoking cessation during treatment were not reported in Duarte’s study. This study also included patients with small-cell lung cancers that have a high sensitivity to CT. Some patients had also received thoracic radiotherapy which was not the case in our study. Finally, the drugs used were a platinum doublet associating cisplatin or carboplatin with etoposide, an association which is no longer in use for the treatment of stage IV NSCLC.

The inconsistency rate between oral declarations and plasmatic cotinine levels was of 35% in our study which is in line with published data [20–22]. Amongst the six patients with inconsistencies, four were women (67%), with a high level of tobacco consumption (median 40 PY, range 30–51). We chose to use cotinine as a marker of continued smoking. Nicotine levels are very specific of cigarette consumption. However, nicotine’s half life is short (two hours) and would only reflect very recent smoking. Cotinine is the principal metabolite of nicotine and is a reliable marker of nicotine exposure. Cotinine can be measured in the blood, plasma or urine. Its half-life is 16 hours and its measurement can reveal cigarette consumption spanning up to three days before the measure. Its specificity with regards to tobacco consumption is close to 100% with a sensitivity of 96 to 97% [23,24].

Molecular mechanisms of platinum-based CT resistance mediated by nicotine are complex. Nicotine plays a direct part in stimulating proliferation. NSCLC cells express the nicotinic acetylcholine receptor at their surface. Nicotine binds to this receptor and leads to the activation of signalling pathways leading to proliferation, survival, angiogenesis and tumor invasion [25]. Activation of the nicotine acetylcholine receptor by nicotine leads to apoptosis escape by cancer cells. It has also been shown that nicotine can induce cancer stem cells proliferation associated with oncogenesis and metastatic spreading [26]. Nicotine can also disrupt platinum salts’ pro-apoptotic action [27–30]. Finally, exposure of cancer cells to nicotine activates the Sonic Hedgehog pathway [26] which is associated with *in vitro* and *in vivo* cisplatin resistance [31,32]. Other studies in the literature support the impact of cigarette smoke on toxicity of cytotoxic chemotherapy [33–35]. However these studies concerned drugs with metabolism induced by cytochrome p450 that is not involved with platinum which has a renal metabolism.

The strength of our study resides on the biological verification of smoking status, allowing us to avoid classification bias caused by false oral declarations. Also, to our knowledge, this is the first study evaluating the impact of continued smoking during CT on tumor response in patients with advanced NSCLC. There are also several limitations: this is an observational, monocentric study with a possible bias during the retrospective part of the study. It is also to be noted that the use of nicotine substitutes can interfere with the plasmatic cotinine levels [36]. We did not, however, find differences regarding plasmatic cotinine levels between patients who declared to use nicotine substitutes and those who did not.

## Conclusion

Continued smoking, evaluated by a biological method (plasmatic cotinine measure), during first-line CT for patients with locally advanced or metastatic NSCLC has a negative impact on ORR. The results from this study need to be confirmed by a prospective study with a larger population.

## Supporting information

S1 File Anonymized study database. (XLSX)
